# 4,4′-Oxybis(2,6-di­methyl­pyridinium) bis­(tri­fluoro­methane­sulfonate)

**DOI:** 10.1107/S1600536813027505

**Published:** 2013-10-12

**Authors:** Amanda W. Stubbs, James A. Golen, Arnold L. Rheingold, David R. Manke

**Affiliations:** a Department of Chemistry and Biochemistry, University of Massachusetts Dartmouth, 285 Old Westport Road, North Dartmouth, MA 02747, USA; b Department of Chemistry, University of California, San Diego, 9500 Gilman Drive, La Jolla, CA 92093, USA

## Abstract

In the asymmetric unit of the title salt, C_14_H_18_N_2_O^2+^·2CF_3_O_3_S^−^, the components are linked by two N—H⋯O and one C—H⋯O hydrogen bonds. The dipyridinium salt demonstrates a skew conformation based upon C—O—C—C torsion angles of 61.5 (3) and 15.1 (4)°. A C—O—C angle of 119.3 (2)° and C—O bond distances of 1.364 (3) and 1.389 (3) Å are consistent with other dipyridyl ethers. The planes of the pyridyl rings exhibit a twist angle of 67.89 (8)°. One of the tri­fluoro­methane­sulfonate ions shows disorder of the F atoms [in a 0.52 (7):0.48 (7) occupancy ratio] and an O atom [0.64 (8):0.36 (8) occupancy ratio]. In the crystal, the components are linked by C—H⋯O inter­actions, which form chains along [101].

## Related literature

For the structure of the unsubstituted 4,4′-oxybisdi­pyridine, see: Dunne *et al.* (1996 ▶). For the structure of bis­[4′-(2,2′:6′,2′′-terpyridin­yl)]ether, see: Constable *et al.* (1995 ▶). For the stuctures of the neutral ether 9,9′-oxybisacridine and its dication, see: Maas (1985 ▶). For a description of conformations in bridged di­phenyls, see: van der Heijden *et al.* (1975 ▶).
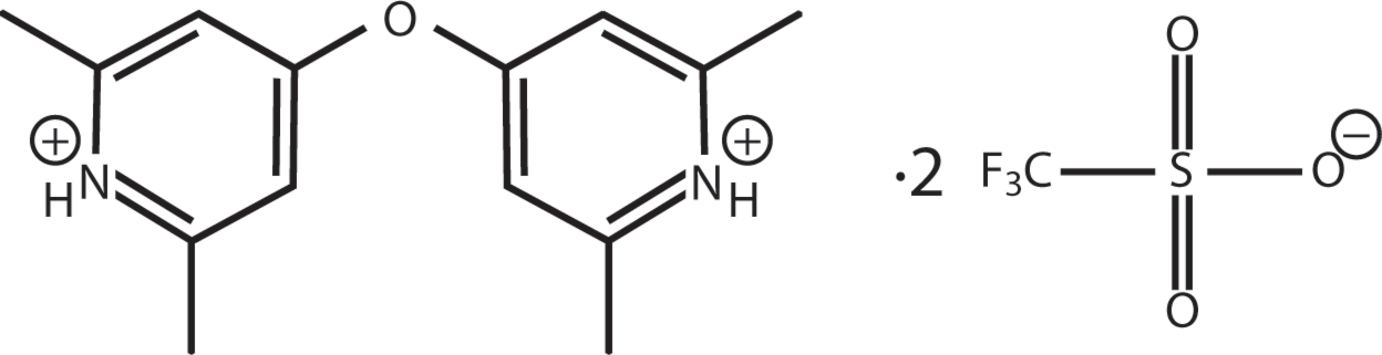


## Experimental

### 

#### Crystal data

C_14_H_18_N_2_O^2+^·2CF_3_O_3_S^−^
*M* *_r_* = 528.44
Monoclinic, 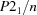

*a* = 12.7397 (18) Å
*b* = 11.3610 (16) Å
*c* = 15.611 (2) Å
β = 101.405 (4)°
*V* = 2214.8 (6) Å^3^
*Z* = 4
Mo *K*α radiation
μ = 0.33 mm^−1^
*T* = 100 K
0.24 × 0.18 × 0.10 mm

#### Data collection

Bruker APEXII CCD diffractometer
Absorption correction: multi-scan (*SADABS*; Bruker, 2005 ▶) *T*_min_ = 0.925, *T*_max_ = 0.968
15390 measured reflections
4360 independent reflections
3546 reflections with *I* > 2σ(*I*)
*R*_int_ = 0.027

#### Refinement

*R*[*F* ^2^ > 2σ(*F* ^2^)] = 0.049
*wR*(*F* ^2^) = 0.125
*S* = 1.09
4360 reflections
316 parameters
53 restraints
H atoms treated by a mixture of independent and constrained refinement
Δρ_max_ = 0.94 e Å^−3^
Δρ_min_ = −1.04 e Å^−3^

### 

Data collection: *APEX2* (Bruker, 2005 ▶); cell refinement: *SAINT* (Bruker, 2005 ▶); data reduction: *SAINT*; program(s) used to solve structure: *SHELXS97* (Sheldrick, 2008 ▶); program(s) used to refine structure: *SHELXL97* (Sheldrick, 2008 ▶); molecular graphics: *SHELXTL* (Sheldrick, 2008 ▶); software used to prepare material for publication: *SHELXTL*.

## Supplementary Material

Crystal structure: contains datablock(s) I, New_Global_Publ_Block. DOI: 10.1107/S1600536813027505/ff2121sup1.cif

Structure factors: contains datablock(s) I. DOI: 10.1107/S1600536813027505/ff2121Isup2.hkl

Supplementary material file. DOI: 10.1107/S1600536813027505/ff2121Isup3.cml

Additional supplementary materials:  crystallographic information; 3D view; checkCIF report

## Figures and Tables

**Table 1:** Hydrogen-bond geometry (Å, °)

*D*—H⋯*A*	*D*—H	H⋯*A*	*D*⋯*A*	*D*—H⋯*A*
N1—H1*N*⋯O4	0.86 (2)	1.93 (2)	2.783 (3)	171 (3)
N2—H2*N*⋯O7	0.87 (2)	1.97 (2)	2.826 (3)	169 (3)
C2—H2*A*⋯O6^i^	0.95	2.36	3.170 (4)	142
C6—H6*B*⋯O6^i^	0.98	2.50	3.383 (4)	149
C7—H7*B*⋯O3^ii^	0.98	2.47	3.421 (4)	164
C9—H9*A*⋯O3^iii^	0.95	2.44	3.293 (4)	149
C12—H12*A*⋯O5^iv^	0.95	2.26	3.168 (4)	160
C14—H14*A*⋯O6	0.98	2.52	3.436 (4)	155

Symmetry codes: (i) 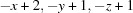; (ii) 
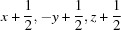
; (iii) 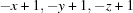; (iv) 
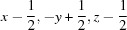
.

**Fig. 1.**
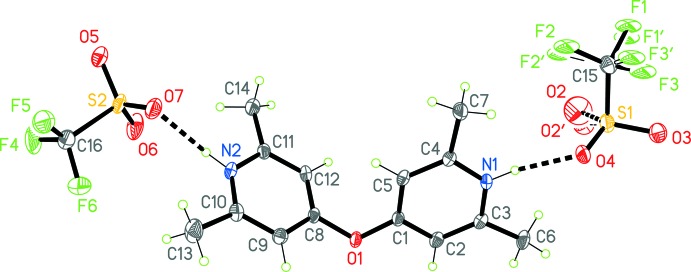
The molecular structure of the title compound, showing the atom labeling scheme, with displacement ellipsoids drawn at the 50% probability level. H atoms are presented as spheres of arbitrary radius. Hydrogen bonding is shown with dashed lines.

## supplementary crystallographic information

### 1. Comment

The structures of bridged diaryls have been examined for many years and here we submit another structure into this data set. Based upon dissimilar C–O–C–C torsion angles of 61.5 (3)° and 15.1 (4)°, this structure exhibits a skew conformation (van der Heijden *et al.* 1975). The previously reported structures of 4,4'-oxybisdipyridyls and their cations (Dunne *et al.* 1996, Maas, 1985, Constable *et al.*, 1995) have shown a twist structure, with torsion angles that are closer in size. Otherwise, the C–O–C angle of 119.3 (2)° and C–O bond distances of 1.364 (3) Å and 1.389 (3) Å are consistent with reported dipyridyl ethers

The structure of the title salt is shown in Figure 1. N–H···O hydrogen bonds between the dication and the two anions are seen between N1–H1N···O4 and N2–H2N···O7. There are no π-π interactions between pyridinium rings of the dications observed. One of the trifluoromethanesulfonate ions shows a disorder at the fluorines with a 52.0:48.0 percentage distribution and at one oxygen with a 64:36 percentage distribution.

### 2. Experimental

Colorless crystals of the title compound formed from the slow decomposition of neat 2,6-dimethyl-4-triflatopyridine.

### 3. Refinement

All non-hydrogen atoms were refined anisotropically by full matrix least squares on F^2^. Fluorine atoms F1, F2, and F3 were disordered over two positions (52.0/48.0) and were refined anisotropically with similar distances and amplitudes using SADI restraints and EADP constraints. Oxygen atom O2 was found to be disordered over two sites (63.5/36.5) and was refined with *DFIX* restraints for S–O bond length of 1.44(0.01) Å and O–O distances of 2.41(0.02) Å and ISOR restraint for O2 and O2'. Hydrogen atoms H1N and H2N were found from a Fourier difference map and were refined isotropically with N—H distance of 0.87 (2) Å and 1.20 *U*_eq_ of parent N atom. All other hydrogen atoms were placed in calculated positions with appropriate carbon hydrogen bond lengths; C—H(Ar) 0.950 Å and CH_3_ 0.980 Å and 1.20 and 1.50 *U*_eq_ of parent C atom.

### Crystal data

**Table:**

C_14_H_18_N_2_O^2+^·2CF_3_O_3_S^−^	*F*(000) = 1080
*M**_r_* = 528.44	*D*_x_ = 1.585 Mg m^−^^3^
Monoclinic, *P*2_1_/*n*	Mo *K*α radiation, λ = 0.71073 Å
Hall symbol: -P 2yn	Cell parameters from 5851 reflections
*a* = 12.7397 (18) Å	θ = 2.4–26.2°
*b* = 11.3610 (16) Å	µ = 0.33 mm^−^^1^
*c* = 15.611 (2) Å	*T* = 100 K
β = 101.405 (4)°	Block, colourless
*V* = 2214.8 (6) Å^3^	0.24 × 0.18 × 0.10 mm
*Z* = 4	

### Data collection

**Table:**

Bruker APEXII CCD diffractometer	4360 independent reflections
Radiation source: fine-focus sealed tube	3546 reflections with *I* > 2σ(*I*)
Graphite monochromator	*R*_int_ = 0.027
φ and ω scans	θ_max_ = 26.0°, θ_min_ = 2.9°
Absorption correction: multi-scan (*SADABS*; Bruker, 2005)	*h* = −15→15
*T*_min_ = 0.925, *T*_max_ = 0.968	*k* = −14→10
15390 measured reflections	*l* = −19→19

### Refinement

**Table:**

Refinement on *F*^2^	Primary atom site location: structure-invariant direct methods
Least-squares matrix: full	Secondary atom site location: difference Fourier map
*R*[*F*^2^ > 2σ(*F*^2^)] = 0.049	Hydrogen site location: inferred from neighbouring sites
*wR*(*F*^2^) = 0.125	H atoms treated by a mixture of independent and constrained refinement
*S* = 1.09	*w* = 1/[σ^2^(*F*_o_^2^) + (0.0486*P*)^2^ + 3.6577*P*] where *P* = (*F*_o_^2^ + 2*F*_c_^2^)/3
4360 reflections	(Δ/σ)_max_ = 0.023
316 parameters	Δρ_max_ = 0.94 e Å^−^^3^
53 restraints	Δρ_min_ = −1.04 e Å^−^^3^

### Special details

**Table:**

Geometry. All e.s.d.'s (except the e.s.d. in the dihedral angle between two l.s. planes) are estimated using the full covariance matrix. The cell e.s.d.'s are taken into account individually in the estimation of e.s.d.'s in distances, angles and torsion angles; correlations between e.s.d.'s in cell parameters are only used when they are defined by crystal symmetry. An approximate (isotropic) treatment of cell e.s.d.'s is used for estimating e.s.d.'s involving l.s. planes.
Refinement. Refinement of *F*^2^ against ALL reflections. The weighted *R*-factor *wR* and goodness of fit *S* are based on *F*^2^, conventional *R*-factors *R* are based on *F*, with *F* set to zero for negative *F*^2^. The threshold expression of *F*^2^ > σ(*F*^2^) is used only for calculating *R*-factors(gt) *etc*. and is not relevant to the choice of reflections for refinement. *R*-factors based on *F*^2^ are statistically about twice as large as those based on *F*, and *R*- factors based on ALL data will be even larger.

### Fractional atomic coordinates and isotropic or equivalent isotropic displacement parameters (Å^2^)

**Table:**

	*x*	*y*	*z*	*U*_iso_*/*U*_eq_	Occ. (<1)
S1	0.30074 (6)	0.21804 (7)	0.30451 (5)	0.0244 (2)	
S2	1.28559 (6)	0.28663 (8)	0.84123 (5)	0.0261 (2)	
F1	0.2582 (5)	−0.0159 (3)	0.3316 (4)	0.0595 (7)	0.520 (7)
F2	0.3592 (5)	0.0635 (5)	0.4423 (4)	0.0595 (7)	0.520 (7)
F3	0.1942 (4)	0.1172 (5)	0.4014 (4)	0.0595 (7)	0.520 (7)
F1'	0.2686 (5)	0.0044 (4)	0.3063 (4)	0.0595 (7)	0.480 (7)
F2'	0.3863 (4)	0.0710 (5)	0.4133 (4)	0.0595 (7)	0.480 (7)
F3'	0.2151 (5)	0.0891 (5)	0.4199 (3)	0.0595 (7)	0.480 (7)
F4	1.45831 (17)	0.3944 (2)	0.92267 (16)	0.0540 (7)	
F5	1.33893 (18)	0.36761 (19)	1.00127 (13)	0.0429 (6)	
F6	1.3158 (2)	0.5016 (2)	0.90176 (19)	0.0642 (8)	
O1	0.81802 (15)	0.55937 (18)	0.51520 (13)	0.0198 (5)	
O2	0.3883 (10)	0.178 (3)	0.268 (2)	0.065 (4)	0.64 (8)
O2'	0.3796 (13)	0.206 (3)	0.2489 (14)	0.039 (5)	0.36 (8)
O3	0.19673 (19)	0.2307 (2)	0.24973 (15)	0.0349 (6)	
O4	0.32838 (16)	0.30914 (19)	0.36998 (14)	0.0247 (5)	
O5	1.33183 (18)	0.1762 (2)	0.87294 (14)	0.0283 (5)	
O6	1.3101 (2)	0.3260 (3)	0.75980 (16)	0.0445 (7)	
O7	1.17400 (17)	0.3012 (2)	0.84615 (16)	0.0322 (6)	
N1	0.53677 (19)	0.3908 (2)	0.42520 (15)	0.0166 (5)	
H1N	0.4755 (19)	0.358 (3)	0.408 (2)	0.020*	
N2	1.05589 (19)	0.4233 (2)	0.70080 (15)	0.0180 (5)	
H2N	1.099 (2)	0.386 (3)	0.7421 (18)	0.022*	
C1	0.7249 (2)	0.4988 (3)	0.48821 (18)	0.0163 (6)	
C2	0.6599 (2)	0.5394 (3)	0.41160 (18)	0.0184 (6)	
H2A	0.6811	0.6052	0.3815	0.022*	
C3	0.5646 (2)	0.4833 (3)	0.38005 (18)	0.0177 (6)	
C4	0.5987 (2)	0.3485 (3)	0.49946 (18)	0.0164 (6)	
C5	0.6942 (2)	0.4040 (3)	0.53340 (18)	0.0167 (6)	
H5A	0.7379	0.3778	0.5865	0.020*	
C6	0.4891 (2)	0.5170 (3)	0.29786 (19)	0.0244 (7)	
H6A	0.4711	0.4472	0.2610	0.037*	
H6B	0.5230	0.5759	0.2664	0.037*	
H6C	0.4237	0.5499	0.3123	0.037*	
C7	0.5605 (2)	0.2412 (3)	0.5387 (2)	0.0255 (7)	
H7A	0.4840	0.2490	0.5389	0.038*	
H7B	0.6003	0.2319	0.5988	0.038*	
H7C	0.5722	0.1721	0.5042	0.038*	
C8	0.8979 (2)	0.5106 (3)	0.57885 (18)	0.0164 (6)	
C9	0.9320 (2)	0.5738 (3)	0.65427 (19)	0.0197 (6)	
H9A	0.9006	0.6475	0.6631	0.024*	
C10	1.0135 (2)	0.5270 (3)	0.71692 (19)	0.0204 (6)	
C11	1.0248 (2)	0.3602 (3)	0.62686 (19)	0.0184 (6)	
C12	0.9443 (2)	0.4049 (3)	0.56316 (19)	0.0178 (6)	
H12A	0.9212	0.3640	0.5097	0.021*	
C13	1.0564 (3)	0.5851 (3)	0.8025 (2)	0.0358 (9)	
H13A	1.0534	0.5298	0.8501	0.054*	
H13B	1.0131	0.6547	0.8088	0.054*	
H13C	1.1308	0.6089	0.8046	0.054*	
C14	1.0780 (3)	0.2449 (3)	0.6199 (2)	0.0268 (7)	
H14A	1.1537	0.2503	0.6483	0.040*	
H14B	1.0725	0.2247	0.5581	0.040*	
H14C	1.0429	0.1839	0.6485	0.040*	
C15	0.2854 (2)	0.0901 (3)	0.3686 (2)	0.0548 (13)	
C16	1.3531 (3)	0.3933 (3)	0.9209 (3)	0.0378 (9)	

### Atomic displacement parameters (Å^2^)

**Table:**

	*U*^11^	*U*^22^	*U*^33^	*U*^12^	*U*^13^	*U*^23^
S1	0.0170 (4)	0.0248 (4)	0.0308 (4)	−0.0001 (3)	0.0029 (3)	−0.0091 (3)
S2	0.0188 (4)	0.0357 (5)	0.0208 (4)	−0.0053 (3)	−0.0038 (3)	0.0133 (3)
F1	0.0720 (13)	0.0293 (11)	0.0614 (17)	−0.0171 (9)	−0.0252 (11)	0.0380 (11)
F2	0.0720 (13)	0.0293 (11)	0.0614 (17)	−0.0171 (9)	−0.0252 (11)	0.0380 (11)
F3	0.0720 (13)	0.0293 (11)	0.0614 (17)	−0.0171 (9)	−0.0252 (11)	0.0380 (11)
F1'	0.0720 (13)	0.0293 (11)	0.0614 (17)	−0.0171 (9)	−0.0252 (11)	0.0380 (11)
F2'	0.0720 (13)	0.0293 (11)	0.0614 (17)	−0.0171 (9)	−0.0252 (11)	0.0380 (11)
F3'	0.0720 (13)	0.0293 (11)	0.0614 (17)	−0.0171 (9)	−0.0252 (11)	0.0380 (11)
F4	0.0295 (12)	0.0722 (17)	0.0517 (14)	−0.0213 (12)	−0.0126 (10)	0.0118 (12)
F5	0.0547 (14)	0.0371 (12)	0.0327 (11)	0.0026 (10)	−0.0012 (10)	−0.0034 (9)
F6	0.0656 (17)	0.0273 (13)	0.084 (2)	−0.0100 (12)	−0.0240 (14)	0.0171 (12)
O1	0.0126 (9)	0.0218 (11)	0.0226 (11)	−0.0023 (8)	−0.0029 (8)	0.0070 (9)
O2	0.050 (4)	0.060 (7)	0.096 (8)	0.011 (4)	0.041 (4)	−0.024 (6)
O2'	0.035 (6)	0.039 (8)	0.047 (8)	0.003 (4)	0.017 (5)	−0.011 (5)
O3	0.0352 (13)	0.0293 (13)	0.0318 (13)	−0.0027 (11)	−0.0139 (10)	−0.0049 (10)
O4	0.0194 (11)	0.0242 (12)	0.0279 (12)	−0.0032 (9)	−0.0019 (9)	−0.0056 (9)
O5	0.0270 (12)	0.0356 (13)	0.0202 (11)	0.0024 (10)	−0.0002 (9)	0.0045 (10)
O6	0.0331 (14)	0.071 (2)	0.0257 (13)	−0.0148 (13)	−0.0037 (10)	0.0244 (13)
O7	0.0202 (11)	0.0377 (14)	0.0354 (13)	−0.0016 (10)	−0.0024 (10)	0.0132 (11)
N1	0.0118 (11)	0.0201 (13)	0.0165 (12)	−0.0003 (10)	−0.0004 (9)	−0.0007 (10)
N2	0.0136 (11)	0.0239 (14)	0.0152 (12)	0.0018 (10)	−0.0004 (9)	0.0021 (10)
C1	0.0118 (13)	0.0184 (14)	0.0181 (14)	0.0011 (11)	0.0013 (11)	−0.0007 (11)
C2	0.0161 (13)	0.0225 (16)	0.0168 (14)	0.0015 (12)	0.0040 (11)	0.0058 (12)
C3	0.0162 (14)	0.0213 (15)	0.0157 (13)	0.0042 (12)	0.0035 (11)	0.0019 (12)
C4	0.0155 (13)	0.0184 (15)	0.0148 (13)	0.0021 (11)	0.0020 (11)	0.0012 (11)
C5	0.0147 (13)	0.0213 (15)	0.0130 (13)	0.0023 (11)	0.0003 (10)	0.0024 (11)
C6	0.0175 (14)	0.0340 (18)	0.0191 (15)	0.0005 (13)	−0.0026 (12)	0.0071 (13)
C7	0.0218 (15)	0.0262 (17)	0.0250 (16)	−0.0054 (13)	−0.0037 (12)	0.0070 (13)
C8	0.0104 (12)	0.0207 (15)	0.0175 (14)	−0.0027 (11)	0.0012 (10)	0.0050 (11)
C9	0.0162 (14)	0.0205 (15)	0.0228 (15)	0.0015 (12)	0.0049 (11)	−0.0001 (12)
C10	0.0172 (14)	0.0264 (16)	0.0172 (14)	−0.0002 (12)	0.0028 (11)	−0.0020 (12)
C11	0.0143 (13)	0.0212 (15)	0.0190 (14)	−0.0019 (12)	0.0012 (11)	0.0005 (12)
C12	0.0138 (13)	0.0236 (16)	0.0150 (13)	−0.0028 (12)	0.0002 (11)	−0.0018 (12)
C13	0.0367 (19)	0.042 (2)	0.0243 (17)	0.0095 (16)	−0.0037 (15)	−0.0122 (15)
C14	0.0226 (15)	0.0240 (17)	0.0303 (17)	0.0034 (13)	−0.0033 (13)	−0.0024 (14)
C15	0.040 (2)	0.025 (2)	0.080 (3)	−0.0091 (18)	−0.033 (2)	0.004 (2)
C16	0.0332 (19)	0.032 (2)	0.041 (2)	−0.0067 (16)	−0.0092 (16)	0.0111 (16)

### Geometric parameters (Å, º)

**Table:**

S1—O2	1.422 (6)	C1—C2	1.392 (4)
S1—O3	1.435 (2)	C2—C3	1.373 (4)
S1—O4	1.448 (2)	C2—H2A	0.9500
S1—O2'	1.459 (9)	C3—C6	1.493 (4)
S1—C15	1.797 (4)	C4—C5	1.380 (4)
S2—O5	1.432 (2)	C4—C7	1.488 (4)
S2—O6	1.439 (2)	C5—H5A	0.9500
S2—O7	1.448 (2)	C6—H6A	0.9800
S2—C16	1.825 (4)	C6—H6B	0.9800
F1—C15	1.352 (4)	C6—H6C	0.9800
F2—C15	1.368 (4)	C7—H7A	0.9800
F3—C15	1.393 (4)	C7—H7B	0.9800
F1'—C15	1.364 (4)	C7—H7C	0.9800
F2'—C15	1.353 (4)	C8—C9	1.374 (4)
F3'—C15	1.314 (4)	C8—C12	1.382 (4)
F4—C16	1.335 (4)	C9—C10	1.384 (4)
F5—C16	1.334 (4)	C9—H9A	0.9500
F6—C16	1.332 (4)	C10—C13	1.493 (4)
O1—C1	1.364 (3)	C11—C12	1.376 (4)
O1—C8	1.389 (3)	C11—C14	1.489 (4)
N1—C3	1.351 (4)	C12—H12A	0.9500
N1—C4	1.354 (4)	C13—H13A	0.9800
N1—H1N	0.86 (2)	C13—H13B	0.9800
N2—C10	1.340 (4)	C13—H13C	0.9800
N2—C11	1.350 (4)	C14—H14A	0.9800
N2—H2N	0.87 (2)	C14—H14B	0.9800
C1—C5	1.386 (4)	C14—H14C	0.9800
			
O2—S1—O3	120.0 (12)	H7A—C7—H7C	109.5
O2—S1—O4	114.2 (5)	H7B—C7—H7C	109.5
O3—S1—O4	114.57 (14)	C9—C8—C12	122.1 (3)
O3—S1—O2'	108.6 (9)	C9—C8—O1	117.9 (3)
O4—S1—O2'	112.7 (8)	C12—C8—O1	119.9 (3)
O2—S1—C15	98.1 (16)	C8—C9—C10	118.1 (3)
O3—S1—C15	102.94 (14)	C8—C9—H9A	121.0
O4—S1—C15	102.87 (14)	C10—C9—H9A	121.0
O2'—S1—C15	114.8 (13)	N2—C10—C9	118.6 (3)
O5—S2—O6	115.54 (17)	N2—C10—C13	117.8 (3)
O5—S2—O7	115.02 (14)	C9—C10—C13	123.6 (3)
O6—S2—O7	113.47 (15)	N2—C11—C12	118.2 (3)
O5—S2—C16	103.86 (15)	N2—C11—C14	118.0 (3)
O6—S2—C16	103.92 (17)	C12—C11—C14	123.8 (3)
O7—S2—C16	102.88 (17)	C11—C12—C8	118.4 (3)
C1—O1—C8	119.3 (2)	C11—C12—H12A	120.8
C3—N1—C4	123.6 (2)	C8—C12—H12A	120.8
C3—N1—H1N	119 (2)	C10—C13—H13A	109.5
C4—N1—H1N	117 (2)	C10—C13—H13B	109.5
C10—N2—C11	124.5 (3)	H13A—C13—H13B	109.5
C10—N2—H2N	120 (2)	C10—C13—H13C	109.5
C11—N2—H2N	114 (2)	H13A—C13—H13C	109.5
O1—C1—C5	123.4 (2)	H13B—C13—H13C	109.5
O1—C1—C2	115.6 (3)	C11—C14—H14A	109.5
C5—C1—C2	121.0 (3)	C11—C14—H14B	109.5
C3—C2—C1	119.2 (3)	H14A—C14—H14B	109.5
C3—C2—H2A	120.4	C11—C14—H14C	109.5
C1—C2—H2A	120.4	H14A—C14—H14C	109.5
N1—C3—C2	118.6 (3)	H14B—C14—H14C	109.5
N1—C3—C6	117.2 (3)	F3'—C15—F2'	112.1 (4)
C2—C3—C6	124.2 (3)	F3'—C15—F1'	113.4 (4)
N1—C4—C5	119.1 (3)	F2'—C15—F1'	104.6 (4)
N1—C4—C7	117.4 (3)	F1—C15—F2	103.8 (4)
C5—C4—C7	123.4 (3)	F1—C15—F3	101.0 (4)
C4—C5—C1	118.4 (3)	F2—C15—F3	102.9 (4)
C4—C5—H5A	120.8	F3'—C15—S1	120.6 (3)
C1—C5—H5A	120.8	F1—C15—S1	122.0 (3)
C3—C6—H6A	109.5	F2'—C15—S1	102.7 (3)
C3—C6—H6B	109.5	F1'—C15—S1	101.5 (3)
H6A—C6—H6B	109.5	F2—C15—S1	121.0 (3)
C3—C6—H6C	109.5	F3—C15—S1	102.5 (3)
H6A—C6—H6C	109.5	F6—C16—F5	107.7 (3)
H6B—C6—H6C	109.5	F6—C16—F4	107.9 (3)
C4—C7—H7A	109.5	F5—C16—F4	107.7 (3)
C4—C7—H7B	109.5	F6—C16—S2	111.2 (2)
H7A—C7—H7B	109.5	F5—C16—S2	111.3 (2)
C4—C7—H7C	109.5	F4—C16—S2	110.9 (3)
			
C8—O1—C1—C5	15.1 (4)	O2'—S1—C15—F3'	−178.5 (8)
C8—O1—C1—C2	−165.8 (3)	O2—S1—C15—F1	−63.4 (9)
O1—C1—C2—C3	−180.0 (3)	O3—S1—C15—F1	60.0 (4)
C5—C1—C2—C3	−0.9 (4)	O4—S1—C15—F1	179.4 (4)
C4—N1—C3—C2	−1.3 (4)	O2'—S1—C15—F1	−57.7 (9)
C4—N1—C3—C6	178.2 (3)	O2—S1—C15—F2'	50.2 (9)
C1—C2—C3—N1	0.6 (4)	O3—S1—C15—F2'	173.7 (4)
C1—C2—C3—C6	−178.8 (3)	O4—S1—C15—F2'	−67.0 (4)
C3—N1—C4—C5	2.1 (4)	O2'—S1—C15—F2'	55.9 (9)
C3—N1—C4—C7	−176.1 (3)	O2—S1—C15—F1'	−57.8 (8)
N1—C4—C5—C1	−2.2 (4)	O3—S1—C15—F1'	65.6 (3)
C7—C4—C5—C1	175.8 (3)	O4—S1—C15—F1'	−175.0 (3)
O1—C1—C5—C4	−179.3 (3)	O2'—S1—C15—F1'	−52.2 (8)
C2—C1—C5—C4	1.6 (4)	O2—S1—C15—F2	71.3 (9)
C1—O1—C8—C9	−121.9 (3)	O3—S1—C15—F2	−165.3 (4)
C1—O1—C8—C12	61.5 (3)	O4—S1—C15—F2	−45.9 (4)
C12—C8—C9—C10	−1.9 (4)	O2'—S1—C15—F2	76.9 (9)
O1—C8—C9—C10	−178.4 (2)	O2—S1—C15—F3	−175.1 (8)
C11—N2—C10—C9	0.1 (4)	O3—S1—C15—F3	−51.7 (3)
C11—N2—C10—C13	179.5 (3)	O4—S1—C15—F3	67.7 (3)
C8—C9—C10—N2	0.7 (4)	O2'—S1—C15—F3	−169.4 (8)
C8—C9—C10—C13	−178.6 (3)	O5—S2—C16—F6	−178.0 (3)
C10—N2—C11—C12	0.3 (4)	O6—S2—C16—F6	60.8 (3)
C10—N2—C11—C14	−178.4 (3)	O7—S2—C16—F6	−57.8 (3)
N2—C11—C12—C8	−1.4 (4)	O5—S2—C16—F5	−57.9 (3)
C14—C11—C12—C8	177.2 (3)	O6—S2—C16—F5	−179.2 (3)
C9—C8—C12—C11	2.3 (4)	O7—S2—C16—F5	62.3 (3)
O1—C8—C12—C11	178.7 (2)	O5—S2—C16—F4	62.0 (3)
O2—S1—C15—F3'	175.9 (9)	O6—S2—C16—F4	−59.3 (3)
O3—S1—C15—F3'	−60.7 (4)	O7—S2—C16—F4	−177.8 (2)
O4—S1—C15—F3'	58.7 (4)		

### Hydrogen-bond geometry (Å, º)

**Table:**

*D*—H···*A*	*D*—H	H···*A*	*D*···*A*	*D*—H···*A*
N1—H1*N*···O4	0.86 (2)	1.93 (2)	2.783 (3)	171 (3)
N2—H2*N*···O7	0.87 (2)	1.97 (2)	2.826 (3)	169 (3)
C2—H2*A*···O6^i^	0.95	2.36	3.170 (4)	142
C6—H6*B*···O6^i^	0.98	2.50	3.383 (4)	149
C7—H7*B*···O3^ii^	0.98	2.47	3.421 (4)	164
C9—H9*A*···O3^iii^	0.95	2.44	3.293 (4)	149
C12—H12*A*···O5^iv^	0.95	2.26	3.168 (4)	160
C14—H14*A*···O6	0.98	2.52	3.436 (4)	155

Symmetry codes: (i) −*x*+2, −*y*+1, −*z*+1; (ii) *x*+1/2, −*y*+1/2, *z*+1/2; (iii) −*x*+1, −*y*+1, −*z*+1; (iv) *x*−1/2, −*y*+1/2, *z*−1/2.
